# Surgical Anatomy

**Published:** 1850-07

**Authors:** 


### ARTICLE III.

*Surgical Anatomy.*—By JOSEPH MACLISE, Surgeon. With Colored Plates. Philadelphia: Lea & Blanchard. Imperial Quarto. (From the Publishers, through S. C. Griggs & Co.)

We have received part first of this large and handsome work. This part contains sixteen large colored plates, and forty pages of descriptive letter-press. The plates, although not of the highest style of art, are accurate and truthful; and there is but one word in the English language descriptive of the letter-press—faultless. "The object of this work is to present to the student of medicine and the practitioner removed from the schools, a series of dissections demonstrative of the relative anatomy of the principal regions of the human body." The work will be issued in four parts; price of the whole, eight dollars. We understand part II. is now ready for delivery. For the quality, it is the cheapest work we have seen, and will constitute a valuable contribution to the surgeon's library. M.

